# A Socioeconomic Analysis of Obesity and Diabetes in New York City

**Published:** 2009-06-15

**Authors:** Jonathan B. Wallach, Mariano J. Rey

**Affiliations:** New York University Medical Center School of Medicine; New York University School of Medicine, New York, New York

New York City is getting healthier by almost all measures, but the twin epidemics of obesity and diabetes are getting worse by the year (1).

— New York City Health Commissioner Thomas R. Frieden, MD, MPH

## The Epidemic of Obesity and Diabetes

Surging obesity rates throughout the United States have rapidly changed the face of diabetes mellitus, spawning a type 2 diabetes epidemic. Whereas in the past the most prevalent form of the disease was type 1, today more than 90% of cases are type 2 (2). From 1991 to 2001, obesity grew nationwide by 74% (2); correspondingly, the prevalence of type 2 diabetes increased 61% during the same period (3). Researchers have calculated that each kilogram increase in body mass increases the risk for developing diabetes by 4.5% (4). Indeed, because of the increasing number of children developing the disease, type 2 is no longer referred to as "adult onset." Because of the obesity epidemic, more than 7.8% of adult Americans have diabetes today (4). The problem is particularly severe in New York City, despite its reputation as a city of fit pedestrians; the prevalence of diabetes among New Yorkers has doubled during the past decade to 12.5%, mirroring surging obesity rates citywide (5). Indeed, uncontrolled diabetes is the leading cause of blindness, end-stage renal disease, and nontraumatic lower-extremity amputations for adult New Yorkers (6).

The somatic consequences of diabetes — including a greatly increased risk of cardiovascular disease, stroke, blindness, renal failure, and amputations — are well-known and documented. Researchers are now also focusing on its devastating effects on mental health, as diabetic New Yorkers are 1.9 times more likely than nondiabetic residents to suffer from depression, anxiety, and other psychological disorders (6).

## Socioeconomics of the Epidemic in New York City

**Figure 1.**
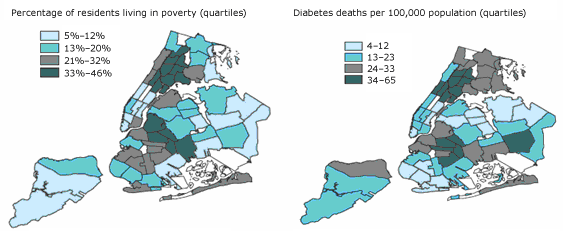
Maps of New York City showing percentages of residents living in poverty and diabetes deaths per 100,000 population, by borough and neighborhood

According to New York City Health Commissioner Thomas R. Frieden, MD, MPH, "Of all diseases New Yorkers suffer, diabetes and HIV have the greatest disparities of race and class" (1). Indeed, throughout the United States, poorer people are more likely to become obese because of factors such as less healthy nutritional habits (healthy foods tend to be more expensive) and lack of time to exercise. They are thus more likely to develop not only obesity but also type 2 diabetes, following a predictable pathway in which poverty leads to obesity, and obesity in turn leads to type 2 diabetes. Furthermore, poorer people are more likely to have severe, uncontrolled disease because of their limited access to health care and health education; regular monitoring and patient self-management reduce diabetes-related morbidity and mortality. Wide income disparities exist in New York City, and the poorest areas (which include the South Bronx, northern Manhattan, and the Brooklyn/Queens border) have the highest levels of obesity and diabetes (Figure 1).

Map at left: Percentages are age-adjusted. Source: US Census 2000/New York City Department of City PlanningMap at right: Rates are age-adjusted. Source: Bureau of Vital Statistics, New York City Department of Health and Mental Hygiene, 2002; US Census 2000/New York City Department of City Planning

**Figure 2.**
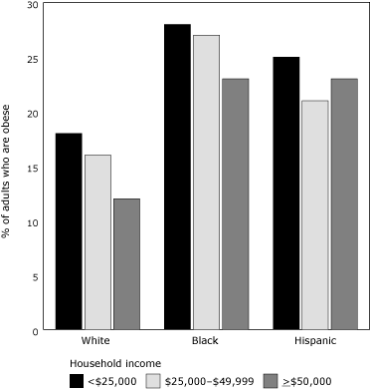
Percentage of New York City residents who are obese and their household income, by racial/ethnic group.

**Figure 3.**
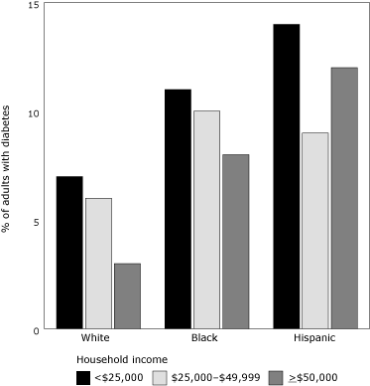
Percentage of New York City residents who are obese and their household income, by racial/ethnic group.

An ethnic component characterizes and contributes to the city's diabesity epidemic, even beyond household income discrepancies. New York City is racially diverse, consisting of about 35% whites, 25% blacks, 27% Hispanics, 10% Asians, and 3% mixed-race or other ethnicity in 2003 (7). Although the entire city's adult population has a diabetes prevalence of 12.5%, it is highest among Asians (16.0%), followed by blacks (14.3%), Hispanics (12.3%), and whites (10.8%). These estimated statistics include undiagnosed cases (5). Racial economic demographics tell us that whites are the least likely to be living in a household with an income less than $25,000 (27%), followed by Asians (32%), blacks (42%), and Hispanics (46%) (7). Yet even among households with similar levels of income, whites have consistently lower rates of obesity and, correspondingly, diabetes than blacks and Hispanics (Figures 2 and 3).

**Table:** 

**Household Income**	**Percent of adults who are obese**		
	**White**	**Black**	**Hispanic**
**< $25,000**	18	28	25
**$25,000 – $49,000**	16	27	21
**> $50,000**	12	23	23

Percentages are age-adjusted.Source: New York City Community Health Survey, 2002. Obesity was defined as body mass index >30 kg/m2, calculated from respondents' height and weight. Complete information was unavailable for Asian household incomes.

**Table:** 

**Household Income**	**Percent of adults with diabetes**		
	**White**	**Black**	**Hispanic**
**< $25,000**	7	11	14
**$25,000 – $49,000**	6	10	9
**> $50,000**	3	8	12

Percentages are age-adjusted.Source: New York City Community Health Survey, 2002. Survey respondents were asked: Have you ever been told by a doctor that you have diabetes? Complete information was unavailable for Asian household incomes.

## Deviations From the Predicted Pathway

The development of diabesity usually follows the predictable pattern in which the poorest income groups are the most likely to be obese and thus develop diabetes. However, for New York City's Hispanics, the trend is unexpectedly U-shaped. Unlike whites and blacks, for Hispanics the highest household income group (≥$50,000) has a higher rate of both obesity and diabetes than does the middle-income group ($25,000-$49,999). This trend occurs even though exercise rates for all ethnic groups consistently increase as household income rises (7). Additional socioeconomic research is needed to elucidate this phenomenon, particularly regarding the dietary habits of the 26% of Hispanic households constituting the wealthiest tier (7).

Asian New Yorkers also deviate from the poverty/obesity/type 2 diabetes pathway when their obesity and diabetes rates are compared with those of other ethnicities. Asian households have incomes slightly below those of white households, and only 5% of adult Asians are obese (far lower than for any other ethnicity) and 24% are overweight (8). Nonetheless, Asians have the highest adult diabetes prevalence (16%) (5). A genetic etiology probably accounts for this discrepancy: a recent major study among women demonstrated that, after adjusting for both age and BMI, compared with whites, blacks were 34% more likely to have diabetes, Hispanics were 86% more likely, and Asians were 126% more likely (9). Even taking into consideration their low BMIs, Asians are still the most likely to develop type 2 diabetes.

## Citywide Efforts to Combat the Epidemic

Health officials are increasingly alarmed by the high rates of obesity and diabetes. In 2002, the municipal government formed the Diabetes Prevention and Control Program with the stated goals of preventing new cases of diabetes, decreasing complications associated with the disease, and increasing quality of life for diabetic residents. Its official 5-point plan consists of professional, community, and patient education; surveillance and evaluation; advocacy; primary prevention of obesity and diabetes; and targeting at-risk youth.

The 2004 New York City Health and Nutrition Examination Survey revealed that more than half of all diabetic adult New Yorkers had hemoglobin A1c levels greater than 7%, indicating poor compliance with recommended self-care measures (5). In 2006, New York City initiated the A1C Registry Program to monitor the blood glucose levels of its diabetic residents, requiring laboratories to report diabetics' hemoglobin A1c results directly to the Health Department. In turn, health officials analyze the data to monitor the quality of care and the extent of the epidemic in various neighborhoods. They are also planning to intervene directly in individual patients' treatments. A program currently being tested in the South Bronx requires city officials to alert doctors to patients who are not adequately controlling their glucose levels. These patients are also personally contacted by city officials and reminded of the medical consequences that can result from poor compliance. Indeed, this program marks the first time any American government has tracked patients who have a chronic, noncommunicable disease (10).

The municipal government is also tightening its regulations on New York City's 22,000 restaurants to promote serving healthier meals. In a nationally publicized move, in July 2007 it limited artificial trans fat content to 0.5 g per serving and issued an additional deadline of July 2008 to stop use of the ingredient entirely. McDonald's and Burger King, the city's largest restaurant chains, have replaced trans fats with somewhat healthier nonhydrogenated soybean, corn, and canola oils and are gradually implementing this change nationally. Philadelphia, Pennsylvania; San Francisco, California; Albany County, New York; and many other governments have followed New York City's lead or are considering the ban.

In February 2006, the city government also began the Gestational Diabetes Initiative to identify and educate mothers who developed gestational diabetes. Studies have shown that approximately 50% of these women will develop diabetes within 6 years, particularly if they are and remain obese (11). This program uses birth certificate records to identify mothers who developed gestational diabetes and then sends resource packets to them describing their potential health risks and how to recognize the symptoms of diabetes. A letter is also sent to tens of thousands of health care providers, reminding them to discuss the risks of gestational diabetes, screen for diabetes before and after delivery, and recommend healthy lifestyle changes.

## Sociodemographic Groups to Target

The municipal government's efforts have been mostly citywide, despite the socioeconomic discrepancies that exist in the diabesity epidemic. To enhance the efficiency and effectiveness of its efforts, New York City needs to begin primary and secondary prevention programs that target populations most at risk.

### Hispanic schoolchildren, particularly boys

**Figure 4.**
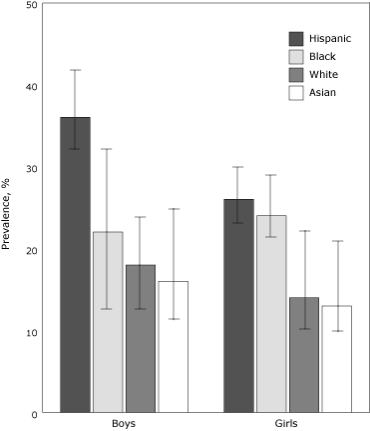
Prevalence (with 95% confidence interval bars) of obesity among New York City public elementary schoolchildren, by sex and race/ethnicity, 2003.

A recent study of 2,681 New York City elementary schoolchildren revealed that 24% are obese and another 19% are overweight (12). Wide discrepancies exist in the prevalence of diabetes among ethnicities and also by sex among Hispanics; Hispanic boys have obesity rates that are significantly higher than those for Hispanic girls (36% vs 26%) (Figure 4) (12). Further aggravating this situation, Hispanic schoolchildren, who make up 40% of the students, have experienced the most rapid rise in obesity (12). These findings necessitate focused intervention with school-based nutritional and physical activity education programs in predominantly Hispanic schools. If such efforts are not undertaken, the 31% obesity rate among Hispanic schoolchildren is likely to cause the current 12.3% diabetes prevalence among Hispanic adults to rise in coming years (5,7).

**Table:** 

	**Prevalence, %**	
	**Boys**	**Girls**
**Hispanic**	36	26
**Black**	22	24
**White**	28	14
**Asian**	26	13

Source: Thorpe LE, List DG, Marx T, May L, Helgerson SD, Frieden TR. Childhood obesity in New York City elementary school students. Am J Public Health 2004;94(9):1496-1500

### Asian communities, particularly schoolchildren

The high diabetes prevalence among Asian New Yorkers is surprising, given their relatively low adult obesity rate of 5% and overweight rate of 24%, and is most likely due to an increased genetic susceptibility at any given BMI. For this reason, Asian communities should be educated about their susceptibility and encouraged to be more vigilant in maintaining a healthy weight and having their blood glucose checked regularly even if they are not overweight.

Particular attention should be focused on educating Asian schoolchildren. In New York City today, an Asian adult obesity rate of 5% correlates with an adult diabetes rate of 16%; because nearly 15% of Asian schoolchildren are obese (3 times the current adult obesity rate), the diabetes rate among Asian adults can be expected to increase as these obese children become adults.

## Conclusions

The New York City government has arguably become the nation's most aggressive municipal government in enacting administrative policies to combat the development and progression of diabetes. Many of its policies, particularly eliminating artificial trans fats from its restaurants, have attracted national headlines and have been copied in other cities. However, diabetes in the city varies widely among different socioeconomic groups. Therefore, more focused efforts need to be undertaken to intervene in the demographic segments at highest risk to tackle more efficiently and effectively the only major health problem that is worsening in the city.
